# Gastric Ulcer

**Published:** 1907-06-01

**Authors:** David Sommerville

**Affiliations:** Lecturer in Public Health, King's College, London; Lecturer in Medical Diseases of the Alimentary Tract, London Polyclinic


### GASTRIC ULCER.

By DAVID SOMMERVILLE, B.A., M.D., M.R.C.P.(Lond.), Lecturer in Public Health, King's College, London; Lecturer in Medical Diseases of the Alimentary Tract, London Polyclinic.

Like the poor, gastric ulcer is always with us. As to its causes, a great deal has been written, much of which has not been based upon experimental evidence. Cruveilhier many years ago described the ulcer, and Rokitansky set down an account of it which the text-books have asserted and re-asserted from that day to the present. It has been described as a round ulcer, a punched-out ulcer, and a perforating ulcer. An important point about it is that it is not due to any single cause. The pathologist who expects to locate this condition on a single basis will be disappointed. The chemical side of the subject is extremely interesting, but I cannot dwell upon that now.

When Virchow discovered embolism he immediately associated it with gastric ulcer. It is a human frailty, more or less universal, for a man who specialises to see too many things through the glasses of his particular work, and for years he may be biased as to the very best work of other research students. Klebs urged arterial spasm as a cause of gastric ulcer. We have at present to recognise the fact that we do not know very much yet about its true cause. The question of the increased secretion of hydrochloric acid lies, I believe, at the root of the whole subject. The ulcer is not produced by the ordinary results of inflammation: that is to say, the reaction brought about by an irritant in throwing out a quantity of blood products is not sufficient in this case, and it is well at the outset to make the distinction that the chemical cause of the condition is not one of proliferation of the various blood and tissue products, but the death of tissue. I think that, without the large increase in the secretion of hydrochloric acid seen in these cases, we could never obtain a gastric ulcer. As to the relation existing between normal tissue and hypersecretion of acid we are no further forward to-day than we were in the days of John Hunter, who 120 years ago explained that the gastric ulcer was produced in those parts of the stomach in which the "vital principle" had failed.

In order to diagnose the condition, and to treat it, one requires some intelligent working formula of etiology. For all practical purposes such a scheme as the following will serve as well as any other: First, one may recognise the condition as produced by impaired vitality due to chemical, thermal, mechanical, or traumatic causes injuring the mucosa. The mechanical impairment has received very little attention compared with the other aspects of the etiology. I have notes of ten cases which indicate that constant repression of the epigastrium has been the starting point of this malady. In these, for a space of several years, no initial symptom referable to gastric ulcer was indicated.

2. Hyperacidity is an all-important factor occurring in conjunction with impaired vitality. It is due to nervous causes, and if the family history be critically examined neuroses of various types may be found for generations before the condition asserts itself. No case of hyperacidity can be found in actual practice apart from such neuroses.

3. Hyperacidity occurs in conjunction with a third etiological factor, namely, altered conditions of blood, quantitative and qualitative, such as are seen in syphilis, anæmia, chlorosis, arteriosclerosis, fatty, amyloid, and aneurysmal degenerations of arteries; and various other conditions including malaria, thrombosis, and embolism.

4. Local bacterial infection. During some years' attendance at the post-mortem room of Guy's Hospital I saw nearly 1,000 autopsies. Of these, 40 presented upon ulcers or scars. Welch concluded on an analysis of 32,000 autopsies that gastric ulcer occurs in 5 per cent. of persons dying from all causes. This same observer has shown that direct bacteriological infection cannot be prevented by an excess of hydrochloric acid in the human stomach.

### Diagnosis.

The symptomatology is often very direct and simple. There is, to start with, pain of a well-defined type, tenderness located to a very small area (often about the size of a florin or half-crown), and generally situated in the epigastrium about the level of the tip of the xiphisternum in the middle line, or slightly to the left of the middle line. Though the gastric wall has no sensory nerves, it is largely supplied with sympathetic fibres which are in relation with certain sensory nerves in the trunk-wall. Thus there are two "pain points," one in front at the spot above mentioned, and one behind at the eleventh dorsal vertebra, and very slightly to the left. Appetite is never lost, and this differentiates ulcer from chronic gastric catarrh, and from certain infective conditions. The patient abstains from food at times simply through sheer fear of the pain that follows food-taking. The appetite remains unimpaired, and as a rule patients do not suffer from indigestion after a liquid meal, but solids, which stimulate the musculature of the stomach and cause peristalsis, elicit very definite pain. Nausea and vomiting very frequently accompany the pain, but not in all cases. The condition of hæmatemesis is not so common as that of pain, nor is melæna. The latter may occur in such small quantity that microscopic and chemical tests are required to detect it. Much has been done by American research on this point, and it is important to be able to employ the new Aloin test, not only in ulcer, but also in dysenteries and other infections of the small and large intestines. There are many conditions upon which light is thrown by the discovery of hæmatin. In cases in which severe hæmatemesis occurs, as also in those in which nausea and vomiting are frequent, loss of weight and secondary anæmia are evident results.

### Differential Diagnosis.

It is necessary to distinguish this disease from hyperchlorhydria. In hypersecretion of acid the pain is not so localised, nor is it so definite in point of time. In hyperchlorhydria the food requires to be swallowed some little time in order to call forth a flow of hydrochloric acid. As the acid increases, so does the pain until in an hour or an hour and a half there is produced a maximum of pain. There is no such curve with the pain of gastric ulcer. Attention has recently been drawn to a method of diagnosis through the introduction of orthoform; in hypersecretion, gastralgia, etc., the orthoform has no effect. In ulcer it is said to relieve pain. Gastralgia in chlorotic women is very difficult at times to diagnose from gastric ulcer. Perhaps the age of the patient may assist slightly in a few cases. Gastric ulcer occurs much more frequently in women under 30 years of age; in men it occurs after 30; various ratios have been set up by different writers.

It is usually not difficult to differentiate gastric ulcer from attacks of gall-stones. In the case of gall-stones we have no specially localised pain, nor any special time of its appearance in relation to a meal; and we have frequently jaundice and a different clinical picture altogether from gastric ulcer, but sometimes the ulcer and the gall-stones may have abnormal symptoms. Both may be latent, and the gastric ulcer may be indicated only by profuse hæmorrhage, and the gall-stone by its discovery in the fæces after an acute attack of pain. Intercostal neuralgia crops up at times, but in this condition the pain is confined to the course of a particular intercostal nerve. I recently saw a case of locomotor ataxia in which the gastric crises were extremely highly developed. At first I was disposed to look upon it as one of gastric ulcer; but the pain was more constant, lasting continuously from two to three days and possessing no relation to the digestion of meals, nor to any precise spot in the epigastrium. From duodenal ulcer one is rarely disposed to diagnose gastric ulcer. I think it is hardly defensible in treatment to use the stomach tube, although sometimes it is serviceable.

### Treatment.

To Cruveilhier we owe the initiation of rational treatment in gastric ulcer. He prescribed physiological rest for the stomach, a small amount of medication, and a modified dietary. Beyond these three points the profession has not as yet got; rest and dietary are the two essentials. I question whether there is much good to be obtained from the administration of drugs. Where hæmatemesis occurs it is absolutely necessary to starve the patient for two or three days. Whether during these two or three days a rectal enema should be administered, depends upon the individual condition of the patient and upon facts relating to the previous history. Perhaps in some cases it is advisable to administer enemata of milk, the peptonisation and preparation of which should be done under the personal inspection of the physician himself. I have spoken previously of the value of dried milk in various forms of gastric disease, and I have now to state that it forms one of the most important food stuffs that can be used in the second and third weeks of the treatment of gastric ulcer. Such a case usually requires to lie up for five or six weeks. Variation in the diet is of considerable import, and the mental effect of adding new materials to the diet daily conduces to a continuous and rapid recovery. It must be remembered that cow's milk forms a dense clot in the stomach, and this is the great argument against its use in gastric ulcer. Dried milk produces no such clot, but a granular precipitate. It is well in these cases to study thoroughly infant dietaries, because, for four or five months, the patient has to live upon such. One should never forget that peptic ulcers are prone to recur either on the old sites of the healed ulcers or in new areas.

